# Preoperative Iron Deficiency Anemia and Frailty in Geriatric Colorectal Cancer Surgery: A Prospective Observational Study

**DOI:** 10.3390/jcm15197642

**Published:** 2026-10-02

**Authors:** Asiye Özkan Kaya, Resul Yılmaz, Şule Arıcan, Ayşe Karakol, Havva Nur Günay, Gülçin Büyükbezirci

**Affiliations:** 1 Department of Anesthesiology and Reanimation, Konya City Hospital, University of Health Sciences, 42020 Konya, Türkiye; 2 Department of Anesthesiology and Reanimation, Faculty of Medicine, Necmettin Erbakan University, 42080 Konya, Türkiye; dr.r.yilmaz@gmail.com (R.Y.); drsulearican@gmail.com (Ş.A.); drgulcin81@gmail.com (G.B.); 3 Department of Anesthesiology and Reanimation, Kastamonu Education and Research Hospital, 37150 Kastamonu, Türkiye; karakolayse@gmail.com; 4 Department of Anesthesiology and Reanimation, Aksaray Education and Research Hospital, 68200 Aksaray, Türkiye; gunay.hnur@gmail.com

**Keywords:** frailty, iron deficiency anemia, colorectal cancer, geriatric anesthesia, postoperative complications

## Abstract

**Objective:** Although iron deficiency anemia (IDA) and frailty are common among geriatric surgical patients—frailty has been reported in approximately 31% of patients undergoing colorectal cancer surgery—prospective data on the association between IDA and frailty in patients undergoing surgery for colorectal cancer remain limited. The primary objective of this study was to compare preoperative frailty, assessed using the total Edmonton Frail Scale (EFS) score, a 17-point multidimensional instrument evaluating cognitive, functional, physical, nutritional and psychosocial domains of frailty, between geriatric patients with and without IDA undergoing elective colorectal cancer surgery. **Methods:** This study included 120 patients aged ≥65 years scheduled for elective laparoscopic colorectal cancer surgery. Preoperative frailty was assessed using the EFS. Anemia was defined as hemoglobin < 13 g/dL; iron metabolism parameters were assessed in anemic patients to determine IDA. The primary outcome was the difference in total EFS scores between patients with and without IDA. Secondary outcomes included early postoperative recovery, postoperative complications, and lengths of intensive care unit (ICU) and hospital stay. **Results:** Anemia was present in 60 patients (50.0%) and IDA in 41 (34.2%). EFS scores were higher in patients with IDA (6.0 [4.5–10.0] vs. 3.0 [2.0–4.0]; *p* < 0.001), and this association persisted after adjustment for age and the Charlson Comorbidity Index (*p* < 0.001). Postoperative complications were more frequent in the IDA group (46.3% vs. 7.6%; *p* < 0.001). In a three-group comparison, anemic patients without iron deficiency did not differ from non-anemic patients in EFS score, Modified Aldrete Score (a 0–10 scale assessing early postoperative recovery in the post-anesthesia care unit) or ICU length of stay, whereas patients with IDA differed from both groups. The EFS discriminated postoperative complications (AUC = 0.807; 95% CI 0.714–0.900), and EFS ≥ 6.5 was associated with complications in the prespecified model (OR 4.51; 95% CI 1.15–17.66; *p* = 0.030), although this was attenuated after adjustment for comorbidity burden (OR 4.64; 95% CI 0.96–22.31; *p* = 0.056). **Conclusions:** Preoperative IDA was associated with greater frailty in geriatric patients undergoing elective colorectal cancer surgery, and this was not explained by anemia alone, since anemic patients without iron deficiency resembled non-anemic patients. Patients with IDA also had more frequent postoperative complications and poorer early recovery. Frailty was associated with postoperative complications, although this was attenuated after adjustment for comorbidity burden. Combined assessment of anemia etiology and frailty may therefore contribute to preoperative risk stratification.

## 1. Introduction

Preoperative identification of patients with reduced physiological reserve and limited capacity to withstand surgical stress remains an important clinical challenge in older adults undergoing surgery for colorectal cancer. Frailty is a multidimensional syndrome characterized by diminished physiological reserve and increased vulnerability to stressors. Anemia, particularly iron deficiency anemia (IDA), is common in this population and represents a potentially identifiable factor that may contribute to frailty. Clarifying the relationship between IDA and frailty may therefore have important implications for the preoperative assessment of older patients undergoing colorectal cancer surgery [1,2].

Among older adults undergoing surgery, frailty represents an important marker of reduced physiological reserve and has been associated with a higher risk of postoperative complications, prolonged hospitalization, intensive care requirement, and mortality. In patients undergoing colorectal surgery, its prevalence has been estimated at approximately 31% [3,4]. Similarly, preoperative anemia has been associated with increased transfusion requirements and adverse postoperative outcomes following major abdominal surgery [5,6]. However, anemia is not a homogeneous condition and can have different clinical implications depending on the underlying etiology. In patients with colorectal cancer, iron deficiency represents an important cause of anemia because of chronic gastrointestinal blood loss, malignancy-associated inflammation, and inadequate nutritional intake. Furthermore, the consequences of iron deficiency may extend beyond reductions in hemoglobin concentration and oxygen-carrying capacity, potentially affecting mitochondrial energy production, skeletal muscle function, and both physical and cognitive performance [7]. The overlap between these effects and the core domains of frailty raises the possibility of a clinically meaningful association between IDA and frailty.

Despite the potential biological overlap between iron deficiency anemia and frailty, prior research has generally considered these conditions independently when examining surgical outcomes. Prospective data directly evaluating the relationship between preoperative IDA and the degree of frailty in older patients with colorectal cancer remain limited. Furthermore, it remains unclear whether the greater frailty burden observed among patients with IDA is attributable primarily to anemia itself or whether iron deficiency identifies a distinct frailty profile among patients with anemia.

In this prospective study, we therefore evaluated the association between IDA and frailty in older patients undergoing surgery for colorectal cancer. Patients with IDA were additionally compared with anemic patients without iron deficiency and with non-anemic patients to determine whether iron deficiency is associated with a distinct frailty profile beyond the presence of anemia alone. This approach was intended to determine whether IDA identifies a clinically relevant phenotype characterized by a greater frailty burden among older surgical patients.

The primary objective of this study was to investigate whether preoperative frailty burden, quantified by the total Edmonton Frail Scale (EFS) score, differed between older patients with and without iron deficiency anemia undergoing elective colorectal cancer surgery. Secondary objectives were to examine the associations of IDA and frailty with early postoperative recovery, postoperative morbidity, and intensive care unit and hospital length of stay, and to evaluate the discriminative performance of the EFS for postoperative complications. We hypothesized that preoperative IDA would be associated with a greater frailty burden. As one of our secondary hypotheses, we further hypothesized that, among patients with anemia, those with IDA would exhibit a greater frailty burden and less favorable postoperative outcomes than those without iron deficiency, indicating that the iron deficiency phenotype rather than anemia alone underlies the association.

## 2. Materials and Methods

### 2.1. Study Design and Ethics

This single-center prospective observational study was conducted at the Department of Anesthesiology and Reanimation at Necmettin Erbakan University Faculty of Medicine Hospital. Patients who met the eligibility criteria between November 2023 and July 2024 were included. This study protocol was approved by the Non-interventional Clinical Ethics Committee, Faculty of Medicine, Necmettin Erbakan University (Date: 16 June 2023; Decision No: 2023/4388). Written informed consent was obtained from all participants. The study was conducted in accordance with the principles of the Declaration of Helsinki and reported following the STROBE guidelines [8].

### 2.2. Participants

Patients were eligible for inclusion if they were aged ≥65 years, had a histologically confirmed colorectal malignancy and were scheduled for elective resection, had an ASA physical status of II–IV, and had sufficient cognitive and communication abilities to provide informed consent. Patients operated on for benign colorectal lesions were not eligible, whereas patients with rectal tumors were included; the cohort therefore comprises colorectal cancer, and the term “colorectal cancer” is used consistently throughout the manuscript. The histological subtype and tumor stage were not recorded in the study dataset. Patients with ASA I physical status were not represented, as individuals free of systemic disease are rarely encountered among geriatric patients scheduled for colorectal cancer surgery. In accordance with the study protocol, the population was restricted to patients undergoing laparoscopic colorectal resection. Exclusion criteria were communication difficulties that precluded adequate assessment, such as hearing or speech impairment, emergency surgery, an open surgical approach planned from the outset, and refusal to participate. Patients who had received red blood cell transfusion or iron replacement therapy within the preceding three months, as well as those with known hematologic disorders or chronic kidney disease, were also excluded. Intraoperative conversion from laparoscopy to open surgery was a prespecified withdrawal criterion: data collection was discontinued at the time of conversion, and the intraoperative and postoperative variables required for the analysis were therefore not obtained in these patients. A total of 120 patients fulfilling these criteria were included in the final analysis.

### 2.3. Study Protocol

Patient demographic characteristics, body mass index, ASA physical status classification, comorbidities, medications and preoperative laboratory parameters were recorded using standardised data collection forms. During preoperative evaluation, the patients’ frailty level was assessed using the Edmonton Frail Scale (EFS). The scale comprises nine domains and eleven parameters: general health status, functional independence, social support, cognitive function, nutrition, medication use, mood, urinary incontinence, and functional performance. Total scores range from 0 to 17, with higher scores indicating a more severe level of frailty. The validity and reliability of the EFS have previously been established in the Turkish population [9,10]. Baseline cognitive status was assessed in all patients during the preoperative period using the Mini-Cog test and the resulting scores were recorded [11]. Comorbidity burden was quantified using the Charlson Comorbidity Index (CCI), calculated from medical records for each patient [12]. As colorectal cancer was the index condition common to all patients, it was not included in the score. In the analyses of the primary outcome, age and the CCI were entered as separate covariates. In the sensitivity model for postoperative complications, age and comorbidity were combined into a single age-adjusted CCI by adding one point per decade of age from 50 years onwards, as proposed for studies with a limited number of outcome events [13]; age was therefore not entered separately in that model.

Serum C-reactive protein (CRP) and serum albumin were obtained from the routine preoperative laboratory panel performed as part of the anesthesia assessment and were retrieved from hospital laboratory records for all patients; they were analysed as markers of systemic inflammation and nutritional status, respectively. In accordance with institutional practice, serum iron, ferritin, transferrin saturation, and total iron-binding capacity measurements were performed only in patients identified with anemia (hemoglobin < 13 g/dL, *n* = 60).

### 2.4. Perioperative Management

All patients received anesthesia according to the institutional standard anesthesia protocol, and all surgical procedures were performed by the same surgical team. Bowel continuity was restored in all patients except the 15 who underwent abdominoperineal resection and received an end colostomy; a coloanal anastomosis was performed in two patients. The anastomotic technique was not recorded in the study dataset. Depth of anesthesia was monitored using the bispectral index (BIS Vista Monitoring System, Medtronic, Minneapolis, MN, USA), and neuromuscular blockade was monitored using train-of-four (TOF -Watch SX, Organon, Oss, The Netherlands) monitoring. Anesthetic agent titration and neuromuscular blockade management were adjusted according to these measurements. Heart rate along with systolic, diastolic, and mean arterial pressures were recorded at eight time points: pre-induction (baseline), post-induction, post-intubation, at 5, 30, and 60 min following surgical incision, at 5 min post-extubation, and at 15 min after admission to the post-anaesthesia care unit (PACU). Duration of surgery, use of vasopressor or inotropic agents, estimated blood loss and transfusion requirements were also recorded. The early recovery was evaluated using the Modified Aldrete Score 15 min after admission to the PACU. Given the observational nature of the study, the research team did not intervene in anesthetic management, choice of medications, fluid therapy, or postoperative care.

### 2.5. Outcomes and Definitions

*Primary Outcome*

The primary outcome was the difference in preoperative frailty burden, quantified using the total EFS score, between patients with and without IDA.

*Secondary Outcomes*

Secondary outcomes included intraoperative hemodynamic profile and management, early postoperative recovery, postoperative complications, and clinical course. For the assessment of perioperative hemodynamics, systolic, diastolic, and mean arterial pressures and heart rate were recorded before induction (baseline), after induction and tracheal intubation, at 5, 30, and 60 min after surgical incision, 5 min after extubation, and 15 min after PACU admission. Intraoperative vasopressor/inotropic requirements, estimated blood loss, and red blood cell transfusion were also recorded.

Early postoperative recovery was assessed 15 min after arrival to the PACU using the Modified Aldrete Score. Postoperative complications that occurred post-operatively and before discharge were classified as cardiovascular, respiratory, or neuropsychiatric. Cardiovascular complications included new arrhythmia, hypertensive crisis, hypotension requiring vasopressor or inotropic support, myocardial ischemia or infarction, and acute heart failure. Respiratory complications included oxygen desaturation, atelectasis, pneumonia, pulmonary edema, respiratory failure, requirement for noninvasive or invasive mechanical ventilation, and reintubation. Neuropsychiatric complications included postoperative delirium screened using the 4AT, agitation assessed using the RASS, and postoperative cognitive change, operationally defined as a decrease of at least 2 points in the Mini-Cog score (score range 0–5) from the preoperative baseline [14,15].

Clinical course outcomes included ICU length of stay, total hospital length of stay, and 30-day mortality.

### 2.6. Definitions of Anemia and Iron Deficiency Anemia

Based on international consensus recommendations for perioperative anemia management, anemia was defined as a hemoglobin concentration < 13 g/dL regardless of sex in patients undergoing major surgery [16].

Iron deficiency was defined as a ferritin concentration < 100 ng/mL and/or transferrin saturation < 20% according to the European Society for Medical Oncology (ESMO) guideline for the management of cancer-associated anemia and iron deficiency [17]. Patients were classified according to the presence of anemia and IDA based on these predefined criteria. As the study was non-interventional, all laboratory parameters were obtained as part of routine clinical care; in line with institutional practice, iron parameters were measured only in patients with anemia. The IDA (−) group therefore comprised non-anemic patients, in whom iron status was not assessed, together with anemic patients in whom iron deficiency was excluded, and denotes the absence of IDA rather than the absence of iron deficiency.

### 2.7. Statistical Analysis

Statistical analyses were performed using IBM SPSS Statistics, version 27.0 (IBM Corp., Armonk, NY, USA). Normality was examined with the Kolmogorov–Smirnov test together with visual inspection of histograms and Q–Q plots and the values of skewness and kurtosis, and variance homogeneity was checked with the Levene test. Continuous data are reported as mean ± standard deviation where normally distributed and otherwise as median with interquartile range; categorical data are given as counts and percentages.

Two independent groups were compared with the independent-samples *t*-test or, for variables departing from normality, with the Mann–Whitney U test. Categorical variables were tested with Pearson’s chi-square test, replaced by Fisher’s exact test wherever an expected cell count fell below five; tables with more than two categories were analysed with the Monte Carlo chi-square test.

Predictors of postoperative complications were first identified in univariable logistic regression; age, sex, body mass index and hemoglobin concentration were then carried into a multivariable model. Collinearity was ruled out by variance inflation factors, none of which exceeded 5. Ferritin was left out of this model because it had been measured only in anemic patients. Model fit is expressed as the Nagelkerke R^2^. A sensitivity model was also fitted in which age and the individual covariates were replaced by the age-adjusted CCI, thereby reducing the number of predictors and raising the events-per-variable ratio. Because the groups differed at baseline in age and comorbidity, the relationship between IDA and the EFS score was re-examined by multiple linear regression with age and the CCI as covariates, both across the whole cohort and within the anemic subgroup; in the latter, body mass index and hemoglobin concentration were added. An extended model further included log-transformed CRP, serum albumin, body mass index and tumor location (right hemicolectomy versus other procedures). The same analysis was repeated after stratification by tumor location, and an IDA-by-location interaction term was fitted. Since the EFS is not normally distributed, every model was repeated on ranks to confirm that the conclusions held. Surgery-related variables were deliberately kept out of these models, as the EFS had been recorded before surgery and cannot be affected by intraoperative events; they were instead compared between subgroups.

To isolate the contribution of iron deficiency, the three groups—patients without anemia, anemic patients without iron deficiency and patients with IDA—were compared directly, using the Kruskal–Wallis test with Dunn’s post hoc procedure for continuous variables and the Monte Carlo chi-square test with pairwise Fisher’s exact tests for categorical variables. All pairwise *p*-values were Bonferroni-corrected.

Discrimination of the EFS for postoperative complications was examined by receiver operating characteristic analysis; the cutoff maximizing the Youden index was taken as optimal, and areas under the curve are reported with 95% confidence intervals. All tests were two-sided, and *p*-values below 0.05 were regarded as statistically significant.

### 2.8. Sample Size Calculation

The sample size was calculated using G*Power version 3.1.9.7 (Heinrich Heine University Düsseldorf, Düsseldorf, Germany) for the primary outcome, namely the comparison of total EFS scores between patients with and without IDA. Because previous data addressing this specific hypothesis were limited, a moderate-to-large effect size (Cohen’s d = 0.70) was assumed. With a two-sided α of 0.05, a power of 95% and equal group sizes, the required sample size was 110 patients (55 per group). Allowing for an anticipated 10% rate of missing or incomplete data, the recruitment target was set at 121 patients. A total of 120 patients fulfilling the eligibility criteria were enrolled; with the group sizes actually obtained (41 with and 79 without IDA), the study retained 95% power to detect the assumed effect size.

## 3. Results

### 3.1. Study Population

**Figure 1:**
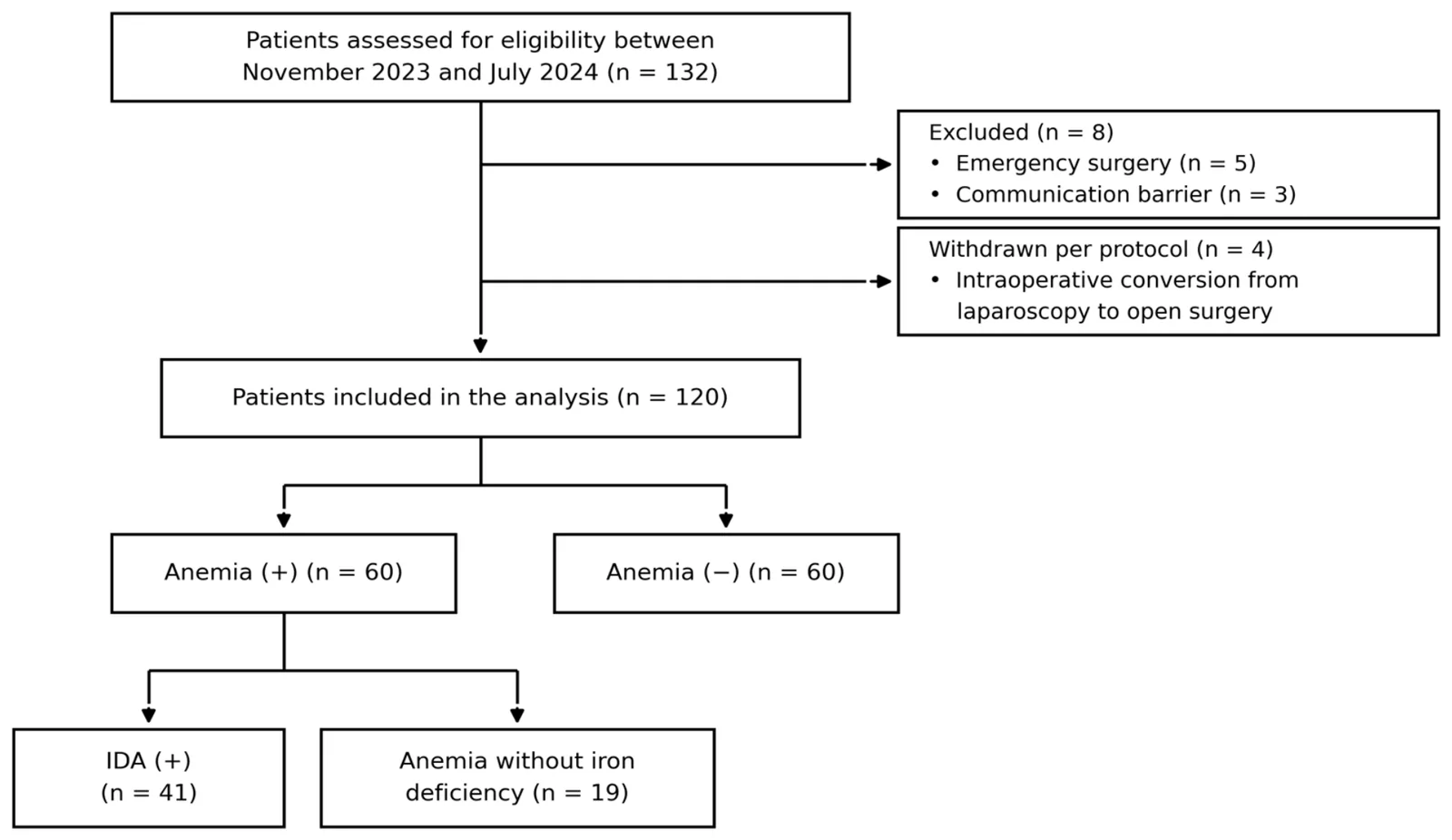
Study Population Flow Diagram.

Between November 2023 and July 2024, 132 patients were assessed, and 120 fulfilled the eligibility criteria (Figure 1). Sixty of them (50.0%) were anemic; iron status was characterized in each, and 41 (68.3%) proved to have IDA, a prevalence of 34.2% across the whole cohort.

### 3.2. Demographic and Clinical Characteristics

The demographic and clinical characteristics of the study population are presented in Table 1. Patients in the IDA (+) group were older (71.0 [67.0–74.0] vs. 68.0 [66.0–71.0] years; *p* = 0.010), and had higher ASA physical status classifications (*p* < 0.001). Hypertension, diabetes mellitus, coronary artery disease, asthma/chronic obstructive pulmonary disease, cerebrovascular disease, and thyroid disease were more prevalent in the IDA (+) group, whereas sex and body mass index did not differ significantly between the groups. Consistent with these differences, the CCI was significantly higher in the IDA (+) group (median 2 [0–3] vs. 1 [0–1]; *p* < 0.001).

Serum albumin was lower in the IDA (+) group (38.8 [35.0–42.0] vs. 40.4 [37.5–44.2] g/L; *p* = 0.025), whereas CRP did not differ significantly (7.8 [3.3–32.5] vs. 5.0 [2.1–15.0] mg/L; *p* = 0.071); hypoalbuminaemia (<35 g/L) was present in 24.4% versus 10.1% of patients (*p* = 0.057). Hemoglobin (10.40 ± 1.25 vs. 13.72 ± 1.38 g/dL) and hematocrit (33.02 ± 3.44% vs. 42.09 ± 3.84%) were significantly lower in the IDA (+) group than in the IDA (−) group (both *p* < 0.001).

In accordance with institutional practice, iron status parameters were assessed only in patients with anemia. Among anemic patients, those with IDA had significantly lower ferritin (15.0 [11.9–35.3] vs. 132.0 [108.0–182.0] ng/mL), serum iron (25.0 [20.1–33.0] vs. 52.0 [43.0–65.0] µg/dL), and transferrin saturation (10.99 ± 4.90% vs. 25.13 ± 5.78%) (all *p* < 0.001), whereas total iron-binding capacity was significantly higher (254.86 ± 60.13 vs. 207.17 ± 51.20 µg/dL; *p* = 0.004). Among the 41 patients with IDA, 40 fulfilled both criteria for absolute iron deficiency (ferritin < 100 ng/mL and transferrin saturation < 20%), and none showed the pattern of functional iron deficiency (ferritin ≥ 100 ng/mL with transferrin saturation < 20%); the median ferritin concentration was 15.0 ng/mL. Conversely, none of the anemic patients classified as iron-replete had a transferrin saturation below 20% (lowest value 21.0%), which argues against appreciable misclassification of iron status by inflammation.

### 3.3. Primary Outcome

The EFS score, the study’s primary outcome, was higher in the IDA (+) group than in the IDA (−) group (6.0 [4.5–10.0] vs. 3.0 [2.0–4.0]; *p* < 0.001) (Table 1). Adjustment for age and the CCI attenuated this difference but did not remove it (unadjusted B = 4.02, 95% CI 3.17–4.88; adjusted B = 2.63, 95% CI 1.96–3.30; *p* < 0.001), and an extended model including log-transformed CRP, serum albumin, body mass index and tumor location left the estimate essentially unchanged (B = 2.54; 95% CI 1.83–3.26; *p* < 0.001). Within the anemic subgroup, where patients without iron deficiency had a markedly lower CCI (median 0 [0–1]), stepwise adjustment attenuated the estimate progressively: from an unadjusted B of 3.92 (95% CI 2.37–5.46) to 1.57 (95% CI 0.41–2.73; *p* = 0.009) after age and the CCI, to 1.25 (95% CI −0.02 to 2.52; *p* = 0.054) once body mass index and hemoglobin were added, the latter to separate iron deficiency from anemia severity, to 1.23 (95% CI −0.07 to 2.53; *p* = 0.063) with CRP and albumin, and to 1.20 (95% CI −0.13 to 2.53; *p* = 0.077) in the fully adjusted model. Rank-based models, used because the EFS is not normally distributed, remained significant at every step (*p* = 0.012 in the fully adjusted model). Surgery-related variables were kept out of these models, since the EFS had been recorded before surgery; they did not differ between the two anemic subgroups (operative duration *p* = 0.521, estimated blood loss *p* = 0.508, right hemicolectomy *p* = 0.218). Tumor location did not account for the association. Adding it to the model adjusted for age and the CCI left the estimate unaltered (B = 2.63; 95% CI 1.95–3.32; *p* < 0.001), and stratification produced effects of similar size in patients undergoing right hemicolectomy (B = 2.61; 95% CI 1.34–3.88) and in those undergoing other procedures (B = 2.73; 95% CI 1.88–3.57), with no interaction (*p* = 0.872). Restricting the analysis to the 54 patients without systemic inflammation (CRP ≤ 5 mg/L) yielded an association at least as strong as in the full cohort (B = 4.18; 95% CI 2.99–5.37; *p* < 0.001

**Figure 2:**
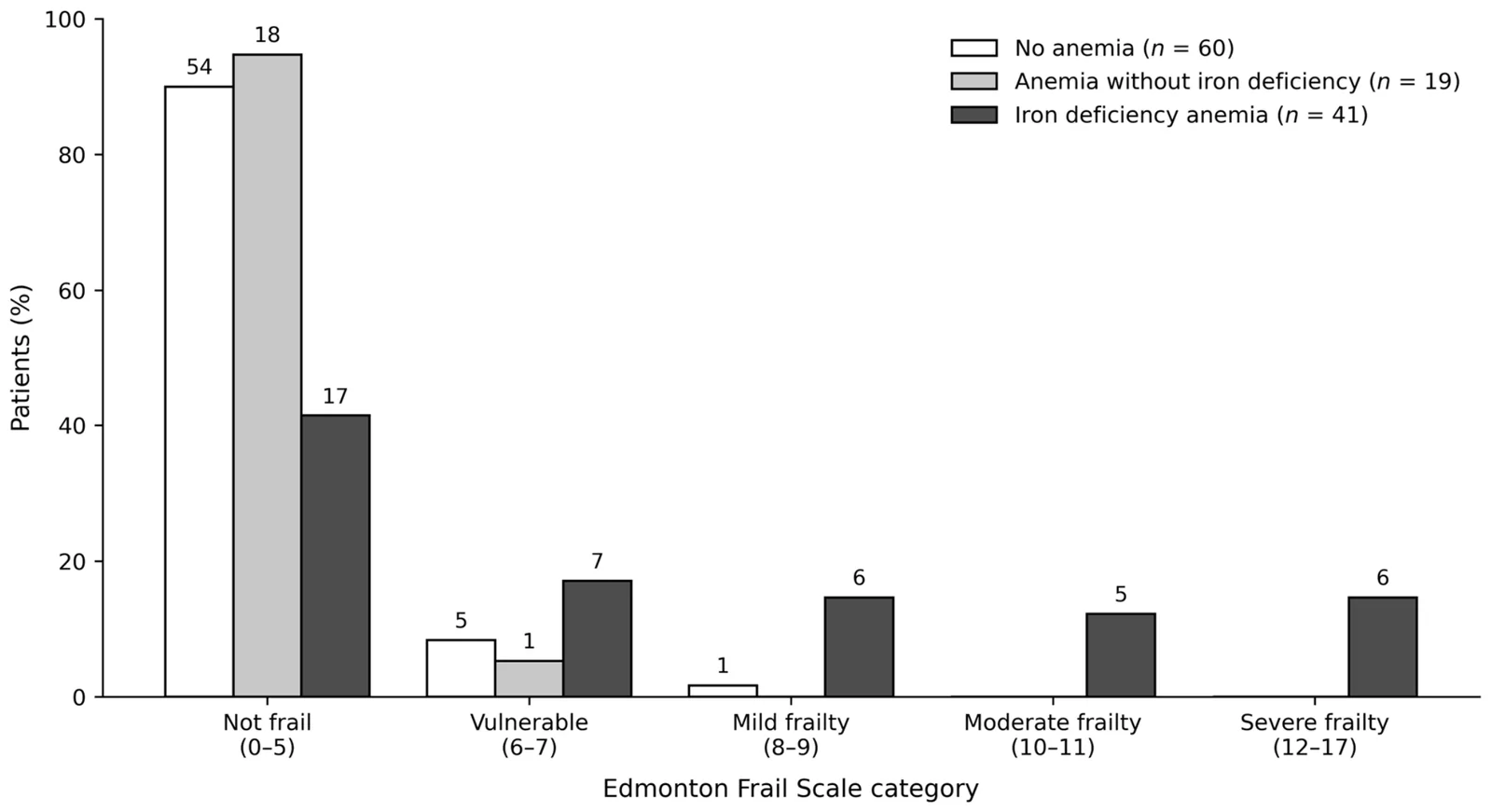
Distribution of Edmonton Frail Scale categories according to anemia and iron deficiency anemia status.

Most patients without anemia (90.0%; *n* = 54) and those with anemia but without iron deficiency (94.7%; *n* = 18) were classified as non-frail, compared with only 41.5% (*n* = 17) of patients with IDA. All patients classified as moderately *(n* = 5) or severely (*n* = 6) frail belonged to the IDA group (Figure 2). Because of low cell frequencies, no formal statistical comparison across frailty categories was performed, and the distribution is presented descriptively.

### 3.4. Secondary Outcomes

Twenty-five patients (20.8%) developed at least one postoperative complication, 32 complications being recorded in total. Complications were far more common when IDA was present (46.3% vs. 7.6%; *p* < 0.001), a pattern that held for each category: cardiovascular (19.5% vs. 3.8%; *p* = 0.008), respiratory (19.5% vs. 3.8%; *p* = 0.008) and neuropsychiatric (22.0% vs. 1.3%; *p* < 0.001) events were all more frequent in the IDA (+) group than in the IDA (−) group (Table 2).

To examine whether these differences were attributable to anemia itself, a formal three-group comparison was performed (Table 3). Anemic patients without iron deficiency did not differ from non-anemic patients in EFS score, Modified Aldrete Score, ICU length of stay or the overall incidence of postoperative complications (all adjusted *p* ≥ 0.236), whereas patients with IDA differed significantly from both groups in each of these outcomes (all adjusted *p* ≤ 0.025). Hospital length of stay, by contrast, was prolonged in both anemic groups compared with non-anemic patients (adjusted *p* = 0.012 and *p* < 0.001, respectively) but did not differ between the two anemic groups (adjusted *p* = 0.136). For individual complication categories the overall differences were significant, but the pairwise differences between the two anemic groups did not remain significant after Bonferroni correction. Operative duration, estimated blood loss and transfusion requirement did not differ between the two anemic groups (*p* = 0.521, *p* = 0.508 and *p* = 1.000, respectively). Because only two anemic patients without iron deficiency developed postoperative complications (one cardiovascular and one respiratory), multivariable adjustment of this comparison was not feasible, and these differences are therefore reported descriptively.

Two patients (1.7%) died within 30 days, both of them in the IDA group; the difference between the IDA (+) and IDA (−) groups did not reach significance (*p* = 0.117), and neither did the three-group comparison (Table 3).

**Figure 3:**
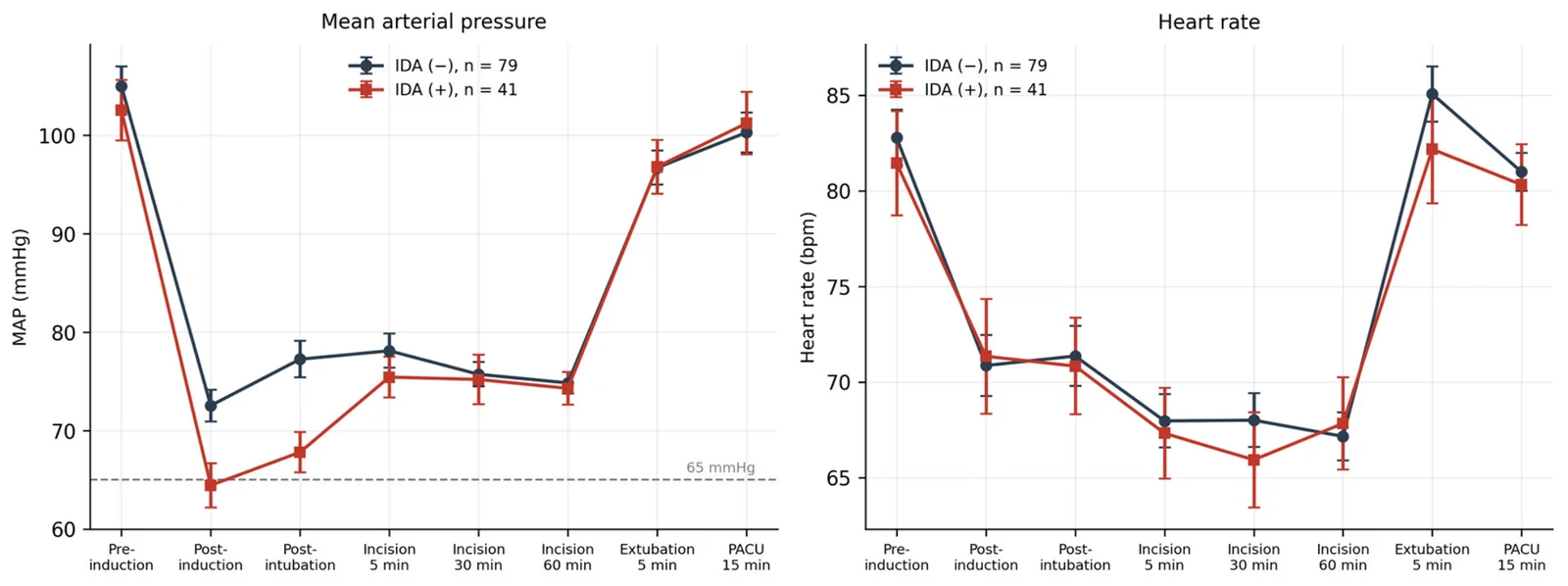
Perioperative course of mean arterial pressure (**left**) and heart rate (**right**) according to the presence of iron deficiency anemia. Values are mean ± standard error. The dashed line indicates the 65 mmHg threshold commonly used to define intraoperative hypotension. IDA, iron deficiency anemia; PACU, post-anesthesia care unit.

Intraoperative management and hemodynamic measurements are shown in Intraoperative management and perioperative hemodynamics are presented in Supplementary Table S1 and Figure 3. Heart rate was comparable between the groups at every time point, as was mean arterial pressure before induction (102.5 ± 19.7 vs. 105.0 ± 17.9 mmHg; *p* = 0.505). Induction changed this: mean arterial pressure fell lower in the IDA (+) group (64.4 ± 14.3 vs. 72.5 ± 14.2 mmHg; *p* = 0.004) and remained lower after tracheal intubation (67.8 ± 13.3 vs. 77.3 ± 16.5 mmHg; *p* < 0.001), with systolic and diastolic pressures behaving the same way. Beyond surgical incision the two groups no longer differed at any time point. Inotropic or vasopressor support was needed more often when IDA was present (22.0% vs. 2.5%; *p* = 0.001), and the two patients (1.7%) who received a red blood cell transfusion both belonged to this group. A post hoc descriptive analysis showed that a mean arterial pressure below 65 mmHg after induction or intubation occurred in 70.7% of patients with IDA against 40.5% of those without (*p* = 0.002); every patient who required vasoactive support fell below this threshold. Since 32 hemodynamic comparisons were made without correction for multiple testing, these analyses should be regarded as exploratory.

### 3.5. Prediction of Postoperative Complications

Receiver operating characteristic analysis identified the EFS as the strongest discriminator of postoperative complications (AUC 0.807; 95% CI 0.714–0.900; *p* < 0.001). The cutoff maximizing the Youden index, an EFS of 6.5 or above, combined a specificity of 91.6% with a sensitivity of 52.0%. Corresponding areas under the curve were 0.778 for ferritin, 0.704 for age and 0.695 for hemoglobin; the ferritin analysis was necessarily confined to the 60 anemic patients in whom iron studies had been performed (Table 4). Univariable logistic regression linked four variables to the occurrence of complications: age (OR 1.13; 95% CI 1.04–1.23; *p* = 0.004), hemoglobin ≤ 12.2 g/dL (OR 5.31; 95% CI 2.01–14.04; *p* < 0.001), ferritin ≤ 49.4 ng/mL, again restricted to anemic patients (OR 10.86; 95% CI 2.22–53.24; *p* = 0.003), and an EFS ≥ 6.5 (OR 11.78; 95% CI 4.05–34.27; *p* < 0.001); neither sex nor body mass index showed any association (Table 5). Once age, sex, body mass index and hemoglobin ≤ 12.2 g/dL were entered together, the EFS threshold alone retained independent predictive value (OR 4.51; 95% CI 1.15–17.66; *p* = 0.030; Nagelkerke R^2^ = 0.307) (Table 6). Replacing age and the individual covariates with the age-adjusted CCI, which reduced the model to three variables and raised the events-per-variable ratio to 8.3, left the effect of an EFS ≥ 6.5 virtually unaltered (OR 4.64; 95% CI 0.96–22.31; *p* = 0.056), while the CCI itself bore no relation to complications (OR 1.17; 95% CI 0.83–1.65; *p* = 0.375). Because the hemoglobin, ferritin and EFS thresholds were derived from the present dataset, the corresponding odds ratios should be interpreted as internally optimistic.

## 4. Discussion

The primary finding of this prospective observational study is that the frailty burden was significantly higher in geriatric colorectal cancer surgery patients presenting with preoperative IDA. In secondary analyses, patients with IDA demonstrated poorer early postoperative recovery, higher complication rates and longer intensive care unit and total hospital stays. Additionally, the EFS demonstrated good discriminatory performance for postoperative complications and maintained its independent association in the prespecified multivariable analysis, although this association was attenuated after adjustment for comorbidity burden.

Frailty is a strong risk determinant in elderly surgical patients, independent of chronological age, associated with postoperative complications, prolonged hospital stays and mortality. In a systematic review encompassing 245,747 patients undergoing colorectal surgery, the prevalence of frailty was reported as 31% and was found to be associated with high complication rates [4]. In elderly cancer patients, this prevalence may reach 58%, with chronic blood loss, malnutrition, inflammation, and tumor-related hypermetabolism identified as the primary determinants. International guidelines recommend the systematic inclusion of frailty assessment in the preoperative process; our findings are consistent with this literature [18,19].

Beyond its role in oxygen transport, iron is essential for mitochondrial energy production and skeletal muscle function; its deficiency has been linked to reduced muscle strength, low physical performance, sarcopenia, and cognitive impairment [7]. A study of 13,175 Chinese adults found each 1 g/dL rise in hemoglobin to lower the odds of frailty by 4% (aOR 0.96; 95% CI 0.93–0.99) [20]. In our cohort, by contrast, anemic patients who were iron-replete showed no detectable excess of frailty relative to patients with normal hemoglobin, although with only 19 such patients a difference in the magnitude implied by that report would have been difficult to detect. Taken together, these observations argue against oxygen-carrying capacity as the sole mechanism.

An important observation of our study is that these differences were not fully accounted for by the presence of anemia alone; this observation, however, does not establish that they are caused by iron deficiency. When patients with IDA were compared separately to anemic patients without iron deficiency, the differences persisted across frailty scores, quality of recovery, intensive care unit length of stay, and the overall incidence of postoperative complications. In a formal three-group analysis, anemic patients without iron deficiency did not differ significantly from non-anemic patients in any of these outcomes. Hospital length of stay was the exception: it was prolonged in both anemic groups, suggesting that this outcome relates to anemia per se rather than to iron deficiency. The distribution of frailty categories demonstrates this pattern even more clearly. Whereas the frailty profile of anemic patients without iron deficiency almost overlapped with that of non-anemic patients, all patients in the moderate and severe frailty categories were in the IDA group. These findings suggest that patients with iron deficiency among those with anemia may constitute a clinical subgroup characterised by a higher frailty burden.

Since patients with IDA were older and more comorbid, we repeated these comparisons with adjustment for age and the Charlson Comorbidity Index. The association held in the cohort as a whole and within the anemic subgroup, attenuating by roughly a third and by more than half respectively. Adding systemic inflammation, nutritional status, body mass index and tumor location to the model shifted the overall estimate only marginally (B = 2.54); in the anemic subgroup, however, the fully adjusted estimate reached only borderline significance in the linear model, remaining significant when the analysis was repeated on ranks.

Part of the difference is therefore attributable to accumulated comorbidity, yet a residual component persists that age and comorbidity do not explain. Notably, the Charlson Comorbidity Index showed no association with postoperative complications while the effect of the EFS survived adjustment intact, which suggests that frailty captures a vulnerability extending beyond the burden of diagnosed disease. Two considerations temper this interpretation: tumor stage and neoadjuvant treatment were not recorded and could not be modelled, and the subgroup comparison is based on a small number of patients. The iron deficiency phenotype is thus best regarded as a marker identifying a higher-risk profile rather than as an established causal determinant of frailty.

Whether preoperative intravenous iron alters clinical outcomes remains unsettled. PREVENTT enrolled patients on the basis of anemia rather than iron status, and ferric carboxymaltose given before elective major abdominal surgery conferred no advantage over placebo in mortality or transfusion requirement, although readmissions fell [6]. Findings of this kind indicate that anemic patients are not a homogeneous population and that the subgroups likely to benefit need clearer definition. Our results suggest the iron deficiency phenotype may be one useful criterion for drawing that distinction.

Discrimination of the EFS for postoperative complications was good (AUC 0.807; 95% CI 0.714–0.900). The threshold identified by the Youden index, an EFS of 6.5 or above, yielded 91.6% specificity but only 52.0% sensitivity; a score below this cutoff therefore cannot be taken to exclude complications, and the threshold itself, derived from the present dataset, requires external validation before it could guide practice. After adjustment for age, sex, body mass index and hemoglobin, an EFS ≥ 6.5 was the only variable that remained independently associated with postoperative complications (OR 4.51; 95% CI 1.15–17.66; *p* = 0.030). The width of this interval reflects how few events were available, and the association weakened to borderline significance when age and the individual covariates were replaced by the age-adjusted Charlson Comorbidity Index (OR 4.64; 95% CI 0.96–22.31; *p* = 0.056)—a pattern consistent with loss of precision rather than loss of effect, but one that leaves confirmation in larger cohorts necessary. The performance of the scale probably owes something to its breadth: it weighs cognition, functional independence, nutrition, medication use, social support and mood alongside physical capacity.

From a clinical perspective, it is of importance that both IDA and frailty are detectable and potentially modifiable conditions in the preoperative period. Assessing these two conditions together may contribute to the early identification of high-risk patients. In such patients, strategies incorporating nutritional support, anemia management, prehabilitation, reduction in polypharmacy, and individualised perioperative monitoring may improve clinical outcomes. Whether targeted preoperative intravenous iron therapy improves outcomes specifically in this high-risk phenotype—frail geriatric patients with IDA—remains unknown. Since PREVENTT enrolled an unselected anemic population, we suggest that future randomized controlled trials should prospectively stratify or restrict enrollment to frail patients with confirmed iron deficiency, using frailty burden alongside perioperative morbidity as endpoints.

The hemodynamic response to anesthetic induction was likewise more marked in patients with IDA: mean arterial pressure approached the conventional 65 mmHg threshold and inotropic or vasopressor support was needed far more often, yet the two groups converged once surgery began. Such a pattern fits reduced physiological reserve rather than a sustained hemodynamic difference, and it places the period of greatest vulnerability squarely in the induction window. Given that even brief intraoperative exposure below 65 mmHg has been associated with acute kidney injury and myocardial injury, this may carry practical implications for anesthetic management in frail patients with IDA, for whom a more cautious induction could be considered.

Deep learning algorithms applied to histopathological images have shown promising accuracy for the classification and diagnosis of colorectal cancer and may, once prospectively validated, shorten the interval between biopsy and definitive diagnosis [21]. A shorter diagnostic interval would in turn widen the window available for preoperative optimization of modifiable conditions such as IDA and frailty. Equally, the effectiveness of screening depends on the knowledge and screening behaviour of healthcare personnel themselves, since gaps in the awareness of nurses and physicians have been identified as a barrier to colorectal cancer screening uptake [22]. Strengthening screening practice among healthcare staff may increase the proportion of tumors detected at an earlier stage, when patients are less likely to present with advanced anemia and accumulated frailty.

Our study has several limitations. Its single-centre design and modest sample size limit how far the findings can be generalised, and with only 25 postoperative complication events the events-per-variable ratio in the multivariable model was low, which widened the confidence intervals.

In line with institutional practice, iron metabolism parameters were measured only in patients with anemia; consequently, non-anemic iron deficiency could not be identified, and the IDA (−) group may have included patients with unrecognized iron deficiency. Such misclassification would be expected to bias the comparison towards the null and therefore to underestimate, rather than exaggerate, the difference between groups. The comparison between patients with IDA and anemic patients without iron deficiency, in whom iron status was fully characterized, is less susceptible to this limitation. Furthermore, although CRP was measured and the iron profile was consistent with absolute iron deficiency in virtually all patients, soluble transferrin receptor and hepcidin were not available; a definitive distinction between absolute and functional iron deficiency therefore rests on ferritin and transferrin saturation alone.

Patients with IDA were significantly older and more comorbid than those without. Since advancing age and accumulating comorbidity raise both frailty and perioperative risk independently, these baseline imbalances are important confounders; age, sex, body mass index and hemoglobin entered the multivariable model and the main comparisons were additionally adjusted for the Charlson Comorbidity Index, yet residual confounding cannot be ruled out. In the sensitivity model for postoperative complications the association of an EFS ≥ 6.5 fell short of conventional significance (*p* = 0.056), reflecting both the moderate correlation between frailty and comorbidity (Spearman ρ = 0.56) and the small number of events.

Tumor location, approximated by the surgical procedure, was taken into account: its distribution differed between the groups, but adjusting for it left the association between IDA and frailty unchanged. Tumor stage and any history of neoadjuvant therapy were not recorded and remain potential confounders, a point of particular relevance to the patients undergoing rectal resection, among whom neoadjuvant chemoradiotherapy is common and may affect both hemoglobin concentration and functional status. Nutritional status was captured only through serum albumin and body mass index; neither the Prognostic Nutritional Index nor formal nutritional screening tools were applied.

Four patients whose procedure was converted from laparoscopy to open surgery were withdrawn in accordance with the protocol, and no outcome data were collected beyond the point of conversion. As conversion is an intraoperative event tied to operative complexity, this withdrawal may have removed higher-risk patients from the cohort and could have produced a modest underestimation of the complication rate; the primary outcome, assessed preoperatively, is unaffected.

The comparison between patients with IDA and anemic patients without iron deficiency was based on only 19 patients in the latter group. This small subgroup markedly limits the statistical power of that comparison, and the inference that the greater frailty burden relates specifically to iron deficiency rather than to anemia per se should therefore be regarded as hypothesis-generating and requires confirmation in larger cohorts.

Finally, preoperative frailty assessment was carried out blinded to laboratory results, but blinding could not be sustained postoperatively; cognitive evaluation relied on the Mini-Cog screening test rather than a comprehensive battery, so the corresponding findings should not be read as a diagnosis of postoperative cognitive dysfunction; and the observational design precludes causal inference, with follow-up extending only to 30 days.

## 5. Conclusions

Among older patients scheduled for elective colorectal cancer surgery, preoperative IDA marked a heavier frailty burden. These patients also sustained more postoperative complications, recovered less well in the early postoperative period and spent longer in intensive care and in hospital. With the exception of hospital stay, these associations held when patients with IDA were compared with anemic patients whose iron status was normal. The iron deficiency phenotype, rather than anemia alone, may therefore identify a higher-risk subgroup, although confirmation is needed in studies able to account for tumor stage and neoadjuvant treatment. Frailty was likewise associated with postoperative complications, an association that weakened once comorbidity burden was taken into account. Assessing the aetiology of anemia alongside frailty during preoperative evaluation may thus improve the identification of older patients at increased perioperative risk; larger multicentre studies are required to confirm these observations.

## Figures and Tables

**Table 1:** Baseline demographic and clinical characteristics according to the presence of iron deficiency anemia.

Variables	Total (*n* = 120)	IDA (−) (*n* = 79)	IDA (+) (*n* = 41)	*p*-Value
Age (years)	68.0 (66.0–73.0)	68.0 (66.0–71.0)	71.0 (67.0–74.0)	0.010 ^2^
Sex (Female), *n* (%)	58 (48.3)	36 (45.6)	22 (53.7)	0.400 ^3^
BMI (kg/m^2^)	27.39 ± 4.69	27.64 ± 4.19	26.92 ± 5.56	0.472 ^1^
ASA II, *n* (%)	62 (51.7)	50 (63.3)	12 (29.3)	<0.001 ^4^
ASA III, *n* (%)	53 (44.2)	29 (36.7)	24 (58.5)	
ASA IV, *n* (%)	5 (4.2)	0 (0.0)	5 (12.2)	
Hypertension, *n* (%)	75 (62.5)	42 (53.2)	33 (80.5)	0.003 ^3^
Diabetes mellitus, *n* (%)	50 (41.7)	25 (31.6)	25 (61.0)	0.002 ^3^
Coronary artery disease, *n* (%)	48 (40.0)	24 (30.4)	24 (58.5)	0.003 ^3^
Asthma/COPD, *n* (%)	32 (26.7)	13 (16.5)	19 (46.3)	0.001 ^3^
Cerebrovascular disease, *n* (%)	14 (11.7)	5 (6.3)	9 (22.0)	0.017 ^5^
Thyroid disease, *n* (%)	24 (20.0)	10 (12.7)	14 (34.1)	0.005 ^3^
Charlson Comorbidity Index, median (IQR)	1 (0–2)	1 (0–1)	2 (0–3)	<0.001 ^2^
C-reactive protein (mg/L)	5.6 (2.6–16.9)	5.0 (2.1–15.0)	7.8 (3.3–32.5)	0.071 ^2^
Serum albumin (g/L)	40.0 (36.9–43.2)	40.4 (37.5–44.2)	38.8 (35.0–42.0)	0.025 ^2^
Hypoalbuminemia (<35 g/L), *n* (%)	18 (15.0)	8 (10.1)	10 (24.4)	0.057 ^5^
Operative duration (min)	140 (120–180)	125 (120–180)	150 (120–215)	0.008 ^2^
Estimated blood loss (mL)	100 (100–200)	100 (100–200)	100 (100–200)	0.427 ^2^
Surgical procedure, *n* (%)				0.007 ^4^
Right hemicolectomy	40 (33.3)	20 (25.3)	20 (48.8)	
Left hemicolectomy/segmental resection	13 (10.8)	10 (12.7)	3 (7.3)	
Sigmoid resection	7 (5.8)	2 (2.5)	5 (12.2)	
Anterior resection of the rectosigmoid	15 (12.5)	13 (16.5)	2 (4.9)	
Low anterior resection of the rectum	24 (20.0)	18 (22.8)	6 (14.6)	
Abdominoperineal resection	15 (12.5)	11 (13.9)	4 (9.8)	
Total colectomy	5 (4.2)	5 (6.3)	0 (0.0)	
Subtotal colectomy	1 (0.8)	0 (0.0)	1 (2.4)	
Edmonton Frail Scale score	4.0 (2.0–6.0)	3.0 (2.0–4.0)	6.0 (4.5–10.0)	<0.001 ^2^

^1^: Independent Samples *t*-test, ^2^: Mann–Whitney U test, ^3^: Pearson chi-square test, ^4^: Monte Carlo chi-square-test, ^5^: Fisher’s Exact test was performed.

**Table 2:** Postoperative Complications According to the Presence of Iron Deficiency Anemia.

Complications	Total (*n* = 120)	IDA (−) (*n* = 79)	IDA (+) (*n* = 41)	*p*-Value
**Any postoperative complication, *n* (%)**	**25 (20.8)**	**6 (7.6)**	**19 (46.3)**	**<0.001 ^1^**
**Cardiovascular complication, *n* (%)**	**11 (9.2)**	**3 (3.8)**	**8 (19.5)**	**0.008 ^2^**
Arrhythmia	2 (1.7)	1 (1.3)	1 (2.4)	
Hypotension	6 (5.0)	2 (2.5)	4 (9.8)	
Hypertension	1 (0.8)	0 (0.0)	1 (2.4)	
Myocardial infarction	1 (0.8)	0 (0.0)	1 (2.4)	
Cardiac Failure	1 (0.8)	0 (0.0)	1 (2.4)	
**Respiratory complication, *n* (%)**	**11 (9.2)**	**3 (3.8)**	**8 (19.5)**	**0.008 ^2^**
Atelectasis	3 (2.5)	1 (1.3)	2 (4.9)	
Hypoxemia	3 (2.5)	1 (1.3)	2 (4.9)	
Pneumonia	1 (0.8)	0 (0.0)	1 (2.4)	
Pulmonary edema	2 (1.7)	1 (1.3)	1 (2.4)	
Requirement for invasive mechanical ventilation	2 (1.7)	0 (0.0)	2 (4.9)	
**Neuropsychiatric complication, *n* (%)**	**10 (8.3)**	**1 (1.3)**	**9 (22.0)**	**<0.001 ^2^**
Postoperative delirium	4 (3.3)	0 (0.0)	4 (9.8)	
Postoperative cognitive change	3 (2.5)	1 (1.3)	2 (4.9)	
Agitation	3 (2.5)	0 (0.0)	3 (7.3)	

^1^: Pearson chi-square test, ^2^: Fisher’s Exact test was performed. Because some patients developed more than one complication, a total of 32 complications occurred in 25 patients; the sum of the category totals therefore exceeds the number of patients with any complication.

**Table 3:** Comparison of Clinical Characteristics, Frailty, and Perioperative Outcomes According to Anemia and Iron Deficiency Status.

Characteristic/Outcome	No Anemia (A) (*n* = 60)	Anemia without ID (B) (*n* = 19)	IDA (C) (*n* = 41)	Overall *p*	A vs. B	A vs. C	B vs. C
Age (years)	68.0 (66.0–71.0)	66.0 (65.0–71.0)	71.0 (67.0–74.0)	0.013	0.728	0.080	0.019
Charlson Comorbidity Index	1 (0–2)	0 (0–1)	2 (0–3)	<0.001	0.058	0.012	<0.001
Hemoglobin (g/dL)	14.1 (13.7–14.9)	11.8 (11.3–12.5)	10.6 (9.4–11.4)	<0.001	<0.001	<0.001	0.139
Edmonton Frail Scale score	3.0 (2.0–4.0)	3.0 (2.0–4.0)	6.0 (4.5–10.0)	<0.001	1.000	<0.001	<0.001
Modified Aldrete Score	9 (9–10)	9 (9–9)	8 (7–8)	<0.001	1.000	<0.001	0.001
ICU length of stay (days)	1 (1–1)	1 (1–2)	2 (2–3)	<0.001	0.236	<0.001	0.023
Hospital length of stay (days)	5 (5–6)	7 (6–8)	8 (7–9)	<0.001	0.012	<0.001	0.136
Any postoperative complication, *n* (%)	4 (6.7)	2 (10.5)	19 (46.3)	<0.001	1.000	<0.001	0.025
Cardiovascular complication, *n* (%)	2 (3.3)	1 (5.3)	8 (19.5)	0.026	1.000	0.042	0.746
Respiratory complication, *n* (%)	2 (3.3)	1 (5.3)	8 (19.5)	0.025	1.000	0.042	0.746
Neuropsychiatric complication, *n* (%)	1 (1.7)	0 (0.0)	9 (22.0)	0.001	1.000	0.003	0.139
30-day mortality, *n* (%)	0 (0.0)	0 (0.0)	2 (4.9)	0.138	1.000	0.487	1.000

Data are presented as median (interquartile range) or *n* (%). A, no anemia; B, anemia without iron deficiency (ID); C, iron deficiency anemia (IDA). Overall *p*-values were obtained with the Kruskal–Wallis test for continuous variables and the Monte Carlo chi-square test for categorical variables. Pairwise *p*-values were obtained with Dunn’s test (continuous variables) or Fisher’s exact test (categorical variables) and adjusted using the Bonferroni correction. ICU, intensive care unit.

**Table 4:** Cut-off values and predictive characteristics in predicting postoperative complications.

	AUC (95% CI)	*p*-Value	Cut-Off ¥	Sensitivity	Specificity
Edmonton Scale	0.807 (0.714–0.900)	**<0.001**	≥6.5	52.0%	91.6%
Age	0.704 (0.599–0.818)	**0.001**	≥72.5	52.0%	80.0%
Hb	0.695 (0.575–0.815)	**0.001**	≤12.20	72.0%	67.4%
Ferritin ^a^	0.778 (0.662–0.894)	**<0.001**	≤49.4	89.5%	56.1%

The development of any postoperative complication (respiratory, cardiovascular or neuropsychiatric) was taken as the endpoint (*n* = 25; %20.8). Patients who did not develop complications served as the reference category. ^a^ As ferritin measurement was performed only in patients diagnosed with anemia, the relevant analysis is limited to the anemic patients (*n* = 60; 19 events); therefore, the resulting AUC value cannot be directly compared with other parameters. ¥ Cut off values determined based on Youden’s J index; AUC: area under the curve; Cl: confidence interval.

**Table 5:** Univariable Logistic Regression Analysis of Factors Associated with the Development of Postoperative Complications.

Univariate Logistic Regression Analysis						
Variables	B	Nagelkerke R^2^	*p* Value	OR	95% CI	
					Lower Bound	Upper Bound
Age	0.125	0.109	**0.004**	1.133	1.041	1.234
Sex	−0.5960	0.022	0.193	0.551	0.225	1.351
BMI	0.024	0.003	0.613	1.024	0.934	1.123
HB (≤12.2)	1.669	0.156	**<0.001**	5.31	2.007	14.042
Ferritin (≤49.4)	2.385	0.264	**0.003**	10.86	2.216	53.244
Edmonton (≥6.5)	2.467	0.259	**<0.001**	11.78	4.050	34.267

Abbreviations: OR = Odds ratio; CI = confidence interval Cut-off values were derived from ROC analysis.

**Table 6:** Multivariable Logistic Regression Analysis of Factors Associated with the Development of Postoperative Complications.

Multivariate Logistic Regression Analysis					
Nagelkerke R^2^ = 0.307					
Variables	B	*p* Value	OR	95% CI	
				Lower Bound	Upper Bound
**Age**	0.073	**0.176**	1.075	0.968	1.195
**Sex**	−0.389	0.467	0.677	0.237	1.936
**BMI**	0.041	0.444	1.042	0.938	1.157
**HB (≤12.2)**	0.958	**0.122**	2.607	0.774	8.776
**Edmonton (≥6.5)**	1.507	**0.03**	4.514	1.154	17.661

Abbreviations: OR = Odds ratio; CI = confidence interval Cut-off values were derived from ROC analysis. Note: The ferritin variable was excluded from the model as it violated the assumption of multicollinearity.

## Data Availability

The data that support the findings of this study are available on request from the corresponding author. The data are not publicly available due to privacy or ethical restrictions.

## Supplementary Materials

The following supporting information can be downloaded at https://www.mdpi.com/article/10.3390/jcm15197642/s1, Table S1: Intraoperative management and perioperative hemodynamic measurements according to the presence of iron deficiency anemia.

## Abbreviations

The following abbreviations are used in this manuscript:

ASA	American Society of Anesthesiologists
AUC	Area under the curve
BMI	Body mass index
CI	Confidence interval
COPD	Chronic obstructive pulmonary disease
EFS	Edmonton Frail Scale
ICU	Intensive care unit
IDA	Iron deficiency anemia
OR	Odds ratio
PACU	Post-anesthesia care unit
ROC	Receiver operating characteristic
STROBE	Strengthening the Reporting of Observational Studies in Epidemiology
