# Supplementary material for: Preoperative Iron Deficiency Anemia and Frailty in Geriatric Colorectal Cancer Surgery: A Prospective Observational Study
Source: J Clin Med. 2026 Oct 2;15(19):7642. doi: 10.3390/jcm15197642 (PMC13666234; doi:10.3390/jcm15197642)
Supplement: Supplementary file 1 [file jcm-15-07642-s001.zip › jcm-4578136-supplementary.pdf]

Table S1: İntraoperative Management And Perioperative Hemodynamic Measurements According To The Presence Of İron Deficiency Anemia.

Variable	Total (n = 120)	IDA (−) (n = 79)	IDA (+) (n = 41)	p-Value
Inotropic/vasopressor support, n (%)	11 (9.2)	2 (2.5)	9 (22.0)	0.001[5]
**Pre-induction (baseline)**				
Heart rate, bpm	82.3 ± 14.6	82.8 ± 12.9	81.4 ± 17.5	0.668[1]
Systolic BP, mmHg	149.5 ± 28.3	149.7 ± 26.9	149.3 ± 31.1	0.946[1]
Diastolic BP, mmHg	77.2 ± 13.7	78.3 ± 12.8	75.0 ± 15.1	0.245[1]
Mean arterial BP, mmHg	104.2 ± 18.5	105.0 ± 17.9	102.5 ± 19.7	0.505[1]
**Post-induction**				
Heart rate, bpm	71.0 ± 16.0	70.9 ± 14.1	71.3 ± 19.2	0.888[1]
Systolic BP, mmHg	98.8 ± 20.7	102.2 ± 19.2	92.2 ± 22.1	0.017[1]
Diastolic BP, mmHg	54.6 ± 12.6	56.4 ± 12.4	51.3 ± 12.4	0.035[1]
Mean arterial BP, mmHg	69.8 ± 14.7	72.5 ± 14.2	64.4 ± 14.3	0.004[1]
**Post-intubation**				
Heart rate, bpm	71.2 ± 14.7	71.4 ± 14.0	70.8 ± 16.2	0.861[1]
Systolic BP, mmHg	106.3 ± 22.7	109.4 ± 22.2	100.5 ± 22.7	0.045[1]
Diastolic BP, mmHg	57.3 ± 14.6	60.1 ± 14.8	51.8 ± 12.5	0.002[1]
Mean arterial BP, mmHg	74.0 ± 16.1	77.3 ± 16.5	67.8 ± 13.3	<0.001[1]
**5 min after incision**				
Heart rate, bpm	67.7 ± 13.4	68.0 ± 12.4	67.3 ± 15.2	0.816[1]
Systolic BP, mmHg	112.0 ± 19.5	113.1 ± 20.9	109.8 ± 16.6	0.354[1]
Diastolic BP, mmHg	59.7 ± 12.5	60.3 ± 12.3	58.7 ± 12.9	0.513[1]
Mean arterial BP, mmHg	77.2 ± 14.6	78.1 ± 15.3	75.4 ± 13.2	0.322[1]
**30 min after incision**				
Heart rate, bpm	67.3 ± 13.7	68.0 ± 12.5	65.9 ± 15.9	0.470[1]
Systolic BP, mmHg	110.3 ± 17.2	110.2 ± 15.3	110.4 ± 20.5	0.962[1]
Diastolic BP, mmHg	58.5 ± 12.1	58.4 ± 9.9	58.8 ± 15.5	0.870[1]
Mean arterial BP, mmHg	75.5 ± 12.9	75.7 ± 11.0	75.2 ± 16.0	0.851[1]
**60 min after incision**				
Heart rate, bpm	67.4 ± 12.7	67.2 ± 11.1	67.8 ± 15.4	0.803[1]
Systolic BP, mmHg	109.2 ± 14.8	110.0 ± 15.0	107.6 ± 14.6	0.389[1]
Diastolic BP, mmHg	57.3 ± 9.4	57.8 ± 8.8	56.4 ± 10.6	0.474[1]
Mean arterial BP, mmHg	74.7 ± 9.9	74.8 ± 9.6	74.3 ± 10.6	0.780[1]
**5 min after extubation**				
Heart rate, bpm	84.1 ± 14.9	85.1 ± 12.9	82.2 ± 18.2	0.368[1]
Systolic BP, mmHg	140.8 ± 24.3	139.8 ± 20.8	142.5 ± 30.2	0.608[1]
Diastolic BP, mmHg	73.5 ± 12.8	73.8 ± 12.7	73.1 ± 13.3	0.808[1]
Mean arterial BP, mmHg	96.7 ± 16.1	96.7 ± 15.4	96.8 ± 17.5	0.976[1]
**15 min after PACU admission**				
Heart rate, bpm	80.8 ± 10.6	81.0 ± 8.8	80.3 ± 13.5	0.775[1]
Systolic BP, mmHg	141.7 ± 25.9	139.5 ± 21.3	145.8 ± 32.8	0.275[1]
Diastolic BP, mmHg	77.4 ± 15.2	77.8 ± 16.0	76.6 ± 13.6	0.667[1]
Mean arterial BP, mmHg	100.6 ± 18.8	100.3 ± 18.1	101.2 ± 20.3	0.806[1]

Data are presented as mean ± standard deviation or n (%). IDA, iron deficiency anemia; PACU, post-anesthesia care unit.

[1] Independent-samples t-test; [5] Fisher's exact test.

Red blood cell transfusion was required in two patients (1.7%), both of whom had IDA; because of the small number of events, this variable was not compared statistically.

Because 32 hemodynamic comparisons were performed, these analyses are exploratory and were not corrected for multiple comparisons.
